# Identification of Autophagy-Related Gene 7 and Autophagic Cell Death in the Planarian *Dugesia japonica*

**DOI:** 10.3389/fphys.2018.01223

**Published:** 2018-09-04

**Authors:** Kexue Ma, Yumei Zhang, Gege Song, Meng Wu, Guangwen Chen

**Affiliations:** College of Life Sciences, Henan Normal University, Xinxiang, China

**Keywords:** planarian, autophagy, autophagic cell death, DjAtg7, regeneration, RNAi

## Abstract

Planarians undergo continuous body size remodeling under starvation or during regeneration. This process likely involves autophagy and autophagic cell death, but this hypothesis is supported by few studies. To test this hypothesis, we cloned and characterized autophagy-related gene 7 (*Atg7*) from the planarian *Dugesia japonica* (*DjAtg7*). The full-length cDNA of *DjAtg7* measures 2272 bp and includes a 2082-bp open reading frame encoding 693 amino acids with a molecular weight of 79.06 kDa. The deduced amino acid sequence of *DjAtg7* contains a conserved ATP-binding site and a catalytic active site of an E1-like enzyme belonging to the ATG7 superfamily. *DjAtg7* transcripts are mainly expressed in intestinal tissues of the intact animals. After amputation, *DjAtg7* was highly expressed at the newly regenerated intestinal branch on days 3–7 of regeneration and in the old tissue of the distal intestinal branch on day 10 of regeneration. However, knockdown of *DjAtg7* by RNAi did not affect planarian regeneration and did not block autophagosome formation, which indicates that autophagy is more complex than previously expected. Interestingly, TEM clearly confirmed that autophagy and autophagic cell death occurred in the old tissues of the newly regenerated planarians and clearly revealed that the dying cell released vesicles containing cellular cytoplasmic contents into the extracellular space. Therefore, the autophagy and autophagic cell death that occurred in the old tissue not only met the demand for body remodeling but also met the demand for energy supply during planarian regeneration. Collectively, our work contributes to the understanding of autophagy and autophagic cell death in planarian regeneration and body remodeling.

## Introduction

Planarians are well known for their extraordinary regenerative ability, and they undergo continuous body size remodeling under starvation or during regeneration (Reddien and Sánchez Alvarado, 2004). After they are transversely cut at the regions before and after the pharynx, the head fragment regenerates a new tail in one week, but the head becomes too large for the newly regenerated planarian. In the following days, the head slowly shrinks and reshapes itself to obtain its proper size. The trunk fragment is also too wide for the length of the newly regenerated planarian, and its pharynx is usually too large. Therefore, the lengthening and thinning of old tissues are important processes during the regeneration of a well-proportioned small planarian. Morgan used morphallaxis to describe body remodeling during planarian regeneration (Morgan, 1900). Tissue remodeling is a fundamental biological process that involves the perfect orchestration of cell differentiation, proliferation, autophagy, and apoptosis. However, the regulation of these processes is poorly understood.

In terms of symmetry and proportion following amputation, many cells are superfluous or incorrectly situated and must be removed via cell death. Two types of programmed cell death, namely apoptotic cell death and autophagic cell death are proposed to be involved in planarian body remodeling. Apoptotic cell death was proposed by Pellettieri et al. (2010), who used a whole-mount terminal deoxynucleotidyl transferase-mediated dUTP nick end labeling (TUNEL) technique and found that cell death increases during planarian body remodeling, but they did not describe the morphological features of cell death. Because TUNEL staining recognizes DNA breaks, the technique can detect not only apoptosis but also late necrosis or autophagic cell death (Nishizaki et al., 1999). Autophagic cell death was proposed by González-Estévez et al. (2007), who found a gene of death-associated protein-1 (*Gtdap-1*), which plays a role in autophagy during planarian regeneration. TEM showed that *Gtdap-1* transcripts are expressed in cells with autophagic morphology, but this work did not provide the morphological features of autophagic cell death (González-Estévez et al., 2007). Notably, planarian regeneration and body remodeling occur under starvation because no food can be taken in until a new functional pharynx is formed. Despite these findings, we have yet to determine exactly how planarians obtain a continuous supply of energy to maintain their life processes during regeneration. One obvious possibility is that autophagy and autophagic cell death provide raw materials for the whole animal’s survival.

Macroautophagy (hereafter referred to as autophagy) is a self-degradation process essential for cell survival under starvation conditions (Meijer and Codogno, 2004; Maiuri and Kroemer, 2015). The classical feature of autophagy involves the double-membrane vesicles that form to sequester a portion of the cytoplasm, long-lived proteins, and intracellular organelles. These double-membrane vesicles, also known as autophagosomes, subsequently fuse with lysosomes to form autolysosomes that degrade their contents for recycling. To date, more than 35 autophagy-related genes (Atgs) have been identified to function at various stages of autophagy (He and Klionsky, 2009; Lee et al., 2015). The formation of the autophagosome requires two ubiquitin-like conjugation systems in which Atg12 is covalently linked to Atg5, and Atg8 is conjugated to phosphatidylethanolamine (Geng and Klionsky, 2008). Atg7, an E1-like ubiquitin, is critical for both conjugation systems. Many reports have revealed that Atg7 is implicated in multiple physiological processes, such as stem cell maintenance (Mortensen et al., 2011; Gomez-Puerto et al., 2016), antioxidative stress (Xie et al., 2015), cell differentiation, and cell proliferation (Karvela et al., 2016). Additionally, the loss of Atg7 function is related to many diseases (Quan et al., 2012; Zhuang et al., 2016; Pu et al., 2017).

Although the mechanism of autophagy has been well studied in many model organisms, it remains poorly investigated in planarians. Additionally, although more than 10 years have lapsed since González-Estévez (2008, 2009) proposed that planarians serve as a new model organism for studies on autophagy, limited research on autophagy has been performed at the molecular level, and no Atg in planarians has been presented. In this work, we focused on the characterization of Atg7 and autophagic cell death during planarian regeneration and body remodeling.

## Materials and Methods

### Animals

Planarians *D. japonica* specimens were collected from Yuquan country (Hebi City, China) and were asexually reproduced in our laboratory. They were reared at 20°C and fed once a week with fresh fish spleen. All animals used for experiments were starved for at least 7 days before the experiments. For regeneration studies, animals were cut at the regions before and after the pharynx.

### RNA Extraction and *DjAtg7* cDNA Cloning

Animals were grinded to powders in liquid nitrogen and immediately extracted using RNAiso Plus reagent (Takara). cDNA was synthesized using PrimeScript 1st strand cDNA synthesis kit (Takara) following the manufacturer’s protocol. Screening our transcriptome data of *D. japonica*, we obtained an EST sequence for *DjAtg7* and designed 3′-RACE specific primer (5′-TGC CAG GTC ATC CAT TGA GTC AGT G-3′) and 5′-RACE specific primer (5′-CTG TAG ATC TAT CCC CGT TAG CTC C-3′) to amplify the full-length cDNA of *DjAtg7*. 5′-RACE and 3′-RACE were performed using a Clontech RACE cDNA amplification kit. The purified PCR products were ligated into the pMD19-T vector and the positive clone was sequenced. The assembled sequence was verified and designated as *DjAtg7*.

### qRT-PCR Analysis

SYBR Green chemistry based qRT-PCR was carried out with ABI PRISM 7500 Sequence Detection System (Applied Biosystems). At each time point, six worms were randomly selected for RNA extraction. Fluorescent real time RT-PCR (qRT-PCR) was performed three times with independent RNA samples. Planarian *elongation factor 2* (*Djef2*) was utilized as the reference gene in all of the experiments (Ma et al., 2017). Expression ratios were determined with the 2^−ΔΔCT^ method, which was described by Livak and Schmittgen (2001). Statistical analysis was performed using SPASS 14.0 as previously described (Li et al., 2013). The data obtained through the qRT-PCR were subjected to one-way ANOVA and differences were considered significant at *P* < 0.05. The following sequence-specific primers were used.

Djef2F: 5′-TTAATGATGGGAAGATATGTTG-3′;
Djef2R: 5′-GTACCATAGGATCTGATTTTGC-3′;
DjAtg7F: 5′-TAACTTTGGAGCTAACGGGGATA-3′;
DjAtg7R: 5′-TACCAACACCGAATAGCAAACAC-3′;
DjAtg1F: 5′-GGTATTTATCGGTCTCGG-3′;
DjAtg1R: 5′-ATGTTGGTGAATCGGTGT-3′;
DjAtg5F: 5′-ATGCTAGTTCCACGAGTTTCG-3′;
DjAtg5R: 5′-AATCGGCTTCTTTCACTGTAT-3′;
DjAtg6F: 5′-GGAACAATTATGGGAGATGC-3′;
DjAtg6R: 5′-AATTCCGCCAGTAAACTCTG-3′;
DjPi3KF: 5′-CTGTTAAAGATGCCAAGACTG-3′;
DjPi3KR: 5′-ATATTATCAGGGTGACGATCTC-3′;
DjAtg8F: 5′-TGGCAGTACAAAGAGGAGCA-3′;
DjAtg8R: 5′-TTAAATCATTGGGAACGAGA-3′;
DjAtg12F: 5′-GGCTGACGATTCAAATGAGG-3′;
DjAtg12R: 5′-AAGTGATGGAGCAAAGGATA-3′;
DjRab9AF: 5′-ATTTAGAGGTTTGGTGGCA-3′;
DjRan9AR: 5′-TTGGCGGATAAAGAAGAA-3′;

### Whole-Mount *in situ* Hybridization (WISH)

Colorimetric whole-mount *in situ* hybridization (WISH) and fluorescent *in situ* hybridization (FISH) were performed as elsewhere described (Umesono et al., 1997; Pearson et al., 2009; Yuan et al., 2014; Han et al., 2018). The following DIG- (Roche) or FITC- (Roche) labeled riboprobes were synthesized using an *in vitro* transcription kit (Roche). The animals were treated with 2N HCl in planarian water for 5 min to clear epidermal mucus, and fixed in 4% formaldehyde in PBSTx for 30 min at RT. Then animals were dehydrated and stored in methanol at −20°C for at least 1 h. The animals were bleached in 6% H_2_O_2_ for 14 h under bright light. The animals were post-fixed for 10 min after treated with 4 μg/ml proteinase K solution. After several washes, animals were incubated in 400 ng/mL Riboprobe mix at 56°C for 16 h. The animals were further incubated in anti-digoxigenin alkaline phosphatase-conjugated antibody (1:2000, Roche), anti-fluorescein-POD (1:1000, Roche) or anti-DIG-POD (1:1000, Roche) overnight at 4°C. For colorimetric development, BCIP/NBT was used as substrates. For fluorescent development, Rhodamine-tyramide and FITC-tyramide were used at 1:1000 and 1:500 separately. For horseradish peroxidase enzyme inactivation, animals were incubated in 154 mmol/L sodium azide for 2 h. Fluorescent images were collected on a Leica TCS SP2 confocal laser scanning microscope and processed in Adobe Photoshop.

### *In vitro* Transcription and RNA Interference (RNAi)

RNAi was performed as previously described (Rouhana et al., 2013; Han et al., 2018). The method for double-stranded RNA (dsRNA) synthesis was performed using the MEGAscript RNAi kit (Ambion, 1626). T7 promoter-containing PCR primers (sense: 5′-TAA TAC GAC TCA CTA TAG GG GCA TTT CCT GCA CCA AGA TTT CCAG-3′ and antisense: 5′-TAA TAC GAC TCA CTA TAG GG CAG CTT TGT TTC CGT CTT TAT GT) were used to amplify the targeted cDNA sequence, and the PCR product was purified as the transcription template. The transcription reaction mixture (1 μg of template DNA, 2 μL of 10X T7 reaction buffer, 8 μL of rNTP mixture, 2 μL of T7 enzyme mix, and nuclease-free water until a final volume of 20 μL is obtained) was incubated at 37°C for 4–6 h and then at 75°C for 10 min, slowly cooled to room temperature to form dsRNA. After treated with RNase-free DNase I to remove the DNA template, 0.5 μL of transcription mixture was run on 1% agarose gel (non-denaturing) to examine the integrity and efficiency of duplex formation. dsRNA was precipitated with 5 M ammonium acetate, and 2 μg of dsRNA was directly fed to the planarians (15–20 young and healthy planarians) by mixing with 20 μL of liver paste. The worms were treated six times for 3 weeks (days 1, 5, 9, 13, 17, and 21). For regeneration studies, planarians were cut at regions before and after the pharynx at 1 day after the last dsRNA feeding was administered. They were subsequently screened with a Leica DFC300FX camera to observe phenotypic changes. RNAi experiments were repeated thrice, and *Dj-β-catenin-1* dsRNA was used as a positive control. RNase-free water instead of dsRNA was used as a negative control. The effect of RNAi was confirmed through qRT-PCR by using a set of primers specific to the targeted genes.

### Transmission Electron Microscopy (TEM)

TEM was performed as previously described (Brubacher, 2014). In brief, the planarians were collected 7 and 10 days after amputation. The new blastema was removed, and the old tissues were immediately fixed in 2.5 % vol/vol glutaraldehyde at 4°C for 24 h. After the samples were washed with PBS, secondary fixation was performed with 1% wt/vol OsO_4_. The samples were dehydrated with a graded alcohol series to 100% and with acetone. Afterward, the samples were embedded in Epon 812 resin mixture. Tissue cross-sections were cut at a thickness of 50–70 nm with an ultramicrotome and stained with uranyl acetate and lead citrate. Ultrathin sections were photographed with a TEM (Hi-7700, Hitachi, Japan). Six samples were randomly selected from each group (the control, R7 days and R10 days, respectively) for TEM.

### Hyperlinking to Databases

Nucleotide sequence and protein sequence data are available in the GenBank databases under the accession number: **KX268473** and **APY27057**.

## Results

### cDNA Cloning and Homology Analysis of DjATG7 in *D. japonica*

**FIGURE 1:**
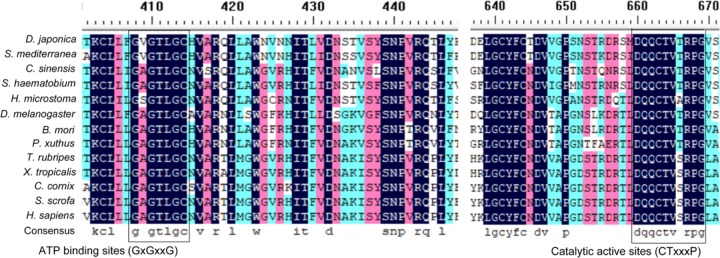
Multi-alignment of ATG7 homologs in invertebrate and vertebrate species. The highly conserved ATP-binding sites (GxGxxG) and catalytic active sites (CTxxxP) are boxed.

The full-length cDNA of *Atg7* from *D. japonica* (*DjAtg7*) is 2272 bp, and it includes a 74-bp 5′-terminal untranslated region (UTR) and a 116-bp 3′-terminal UTR. The open reading frame (ORF) of *DjAtg7* measures 2082 bp and encodes a polypeptide of 693 amino acids with a 79.06 kDa predicted molecular mass and a theoretical isoelectric point of 6.66 (GenBank Accession No. **KX268473**). NCBI blast showed that DjATG7 amino acid sequence is 41–46% identical to various animal-derived ATG7 sequences: 46.41% in *Clonorchis sinensis*, 40.72% in *Drosophila melanogaster*, 42.57% in *Xenopus tropicalis*, 41.88% in *Sus scrofa*, and 41% in *Homo sapiens*. Multi-alignment showed that two highly conserved domains of E1-activating enzymes—ATP-binding sites (GxGxxG domain) and catalytic active sites (CTxxxP domain)—are both present in the ATG7 homologs of invertebrate and vertebrate species (**Figure 1**).

**FIGURE 2:**
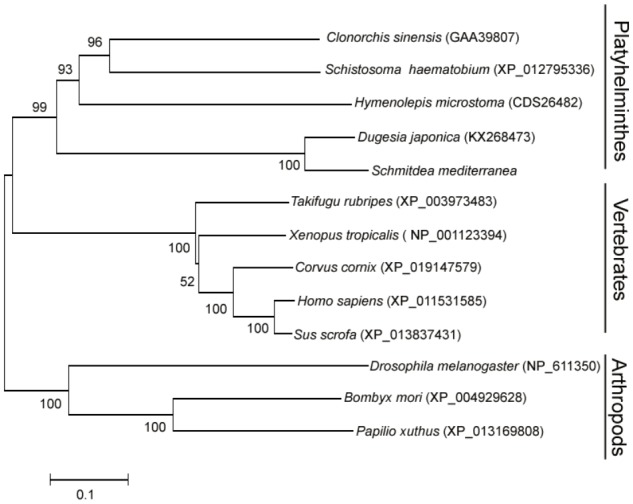
The phylogenetic tree of the ATG7 protein family, constructed with the neighbor-joining method. The number on each branch indicates the confidence percentage from a bootstrap test of 1000 pseudoreplicates. GenBank accession numbers are in brackets.

To elucidate the evolutionary position of DjATG7, we constructed a phylogenetic tree for ATG7 via the neighbor-joining (NJ) method. The phylogenetic tree showed that all Platyhelminthes species were clustered to form one branch, and its sister branch included vertebrate species (**Figure 2**). Two planarian species were clustered with a boot strap percentage of 100%. The tree further showed that planarians are more closely related to vertebrates than to arthropods. Early studies reported that some nervous system related genes are shared by planarians and humans but are absent in *D. melanogaster* (Mineta et al., 2003). These data support the description of a planarian with a new taxonomy proposed by Carranza et al. (1997).

### Expression Pattern of *DjAtg7* During Planarian Regeneration

**FIGURE 3:**
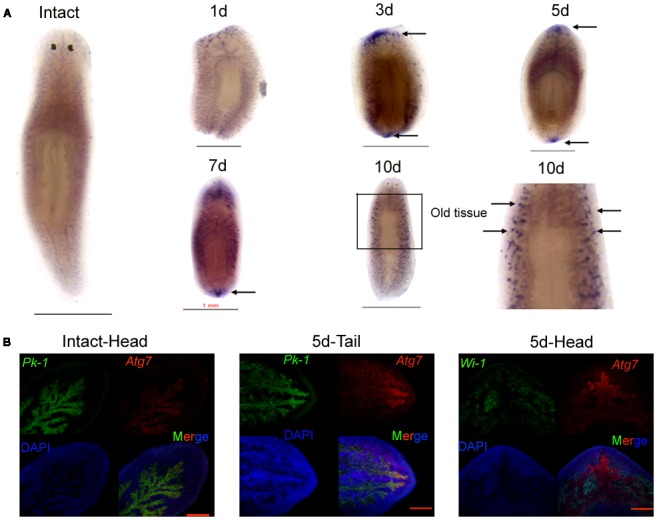
Expression pattern of *DjAtg7* during planarian regeneration. **(A)** Colorimetric WISH in intact and regenerating animals. Intense staining signals are indicated by black arrows. For each time point, *n* = 10 animals. Scale bars: 1 mm. Colorimetric WISH was repeated three times. **(B)** Double FISH of *DjAtg7* (red) and *DjPk-1/DjWi-1* (green) with DAPI (blue). *n* = 6 animals. Scale bars: 200 μm.

We employed qRT-PCR to detect the dynamic changes in *DjAtg7* expression on various days following amputation and to understand whether *DjAtg7* is involved in planarian regeneration and body remodeling. Our results show that *DjAtg7* transcripts increased by 1.33-fold on day 3 of regeneration and maintained relatively high levels on days 5, 7, and 10 of regeneration (1.45-fold, 1.52-fold, 1.64-fold, respectively) (**Supplementary Figure S1**). To further analyze the spatial expression of *DjAtg7*, we used WISH and double-FISH in intact and regenerating animals. In the intact animals, *DjAtg7* transcripts were mainly expressed in intestinal tissues (**Figure 3A**). In the regenerating animals, intense staining signals appeared on the blastema after 3–7 days of regeneration (**Figure 3A**). The colocalization of *DjAtg7* and *DjPk*-1(**Figure 3B** and **Supplementary Figure S2**), a specific intestine marker (Ma et al., 2010), further confirmed that *DjAtg7* is mainly expressed in intestinal tissues and is highly expressed in the newly regenerated intestinal branch. Additionally, the *DjAtg7* signals were not merged with *DjWi-1* (a specific neoblast marker, Rossi et al., 2006) signals in the blastema (**Figure 3B** and **Supplementary Figure S2**), suggesting that the newly regenerated intestinal cells are not derived from neoblasts and/or immediate neoblast descendants. After 10 days of regeneration, intense signals disappeared from the blastema but appeared on the distal branch of the intestine in the old tissues (**Figure 3A**). The expression pattern of *DjAtg7* indicates that the gene plays a role in intestine regeneration during the early phase of planarian regeneration and in body remodeling during the late phase of planarian regeneration.

### Identification of Autophagy and Autophagic Cell Death in Planarian Body Remodeling

**FIGURE 4:**
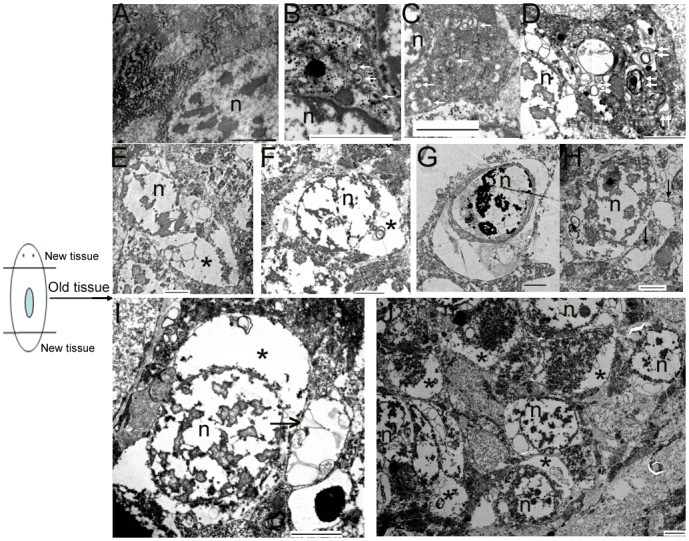
Morphological features of autophagy and autophagic cell death during planarian body remodeling. **(A)** Capture of an intact, normal cell. **(B–G)** The morphological features of each stage of autophagy and autophagic cell death. **(H)** A vesicle being released to the outside of the cell through the plasma membrane. **(I)** A large vesicle containing cellular cytoplasmic contents being released to the outside of the cell. **(J)** Lower magnification showing many cells with the emptied cytoplasms. The white arrow indicates the autophagic vesicles. The double white arrow indicates autophagosomes with multiple membranes. The black arrow indicates the released vesicles containing cellular cytoplasmic contents. The star indicates the emptied cytoplasm. n, nucleus; Scale bar: 2 μm.

To verify whether autophagy and autophagic cell death occurs in the old tissues during planarian body remodeling after amputation, we collected regenerating animals on days 7 and 10 for TEM. In the normal cells, the nucleus remained round, and no autophagic vesicles existed in the cytoplasm (**Figure 4A**). In the early stage of autophagy, autophagic vesicles began to appear in the cytoplasm (**Figure 4B**). In the middle stage of autophagy, numerous autophagic vesicles accumulated in the cytoplasm (**Figure 4C**) and transformed into large, multilayered, membrane-bound structures previously described as autophagosomes (Lee and Baehrecke, 2001; Inbal et al., 2002; Levine and Yuan, 2005; Nishida et al., 2009; Eickel et al., 2013; **Figure 4D**). In the late stage of autophagy, the cell exhibited the classical features of autophagic cell death; that is, the cytoplasm was auto-digested except for the nucleus (**Figures 4E,F**). In the final stage of autophagy, the chromatin was highly condensed and degraded into fragments but was still surrounded with multilayered membranes (**Figure 4G**). Interestingly, we observed that large vesicles containing small vesicles were released from the inside to the outside of the cell (**Figures 4H,I**). This phenomenon is interpreted as the emptying of the cytoplasms occurred in many cells (**Figures 4E,F,H–J**) and is also consistent with the hypothesis that autophagic cell death (a type of selective cell death in planarian) can provide nutrient materials for the survival of other cells under starvation conditions.

### Effects of RNAi-*DjAtg7* on Planarian Regeneration and Body Remodeling

**FIGURE 5:**
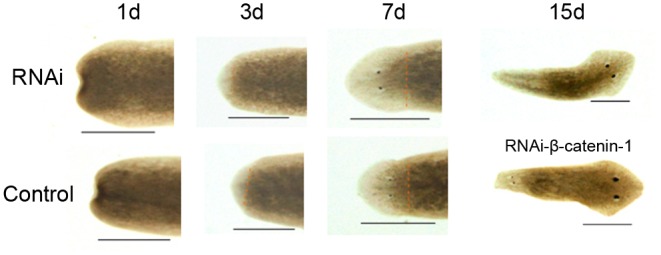
Effects of RNAi-*DjAtg7* on planarian regeneration. The dashed brown line represents the border between blastema and old tissue. RNAi experiments were repeated three times. Scale bar: 0.5 mm.

**FIGURE 6:**
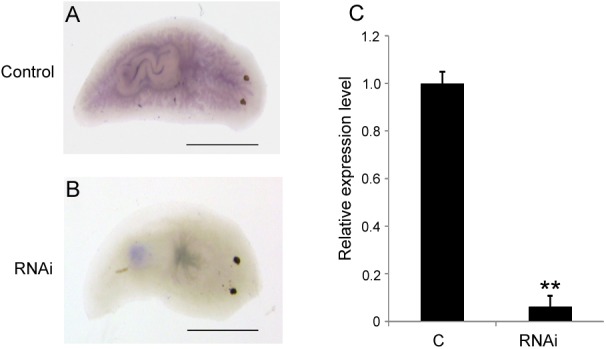
Detection of *DjAtg7* expression in RNAi-animals by WISH and qPCR. **(A)** the control animals, *n* = 10. **(B)** RNAi animals, *n* = 10. **(C)** qPCR shown are averages of three independent experiments; error bars = SEM, ^∗∗^*P* < 0.01 (Student’s *t*-test). Asterisks indicate significant differences compared to the control. Samples were collected at day 10 of regeneration. Scale bar: 0.5 mm.

To investigate the biological function of *DjAtg7* in planarian regeneration, we inhibited *DjAtg7* expression through RNAi. The RNAi-*DjAtg7* animals showed normal regeneration (**Figure 5**). After 15 days of regeneration, a head fragment could regenerate a normal tail and gradually reshape its size to a well-proportioned small animal (**Figure 5**, 72/72). We further used WISH to reveal *DjAtg7* transcripts but found no apparent positive signals in RNAi-animals (**Figure 6B**). We also performed qRT-PCR to detect endogenous *DjAtg7* expression. The results revealed that endogenous *DjAtg7* expression was significantly lower in RNAi-animals (0.045-fold) than in the control animals (**Figure 6C**). As expected, the positive control animals could regenerate a new head in the location where tail regeneration occurred in the RNAi-*Dj-β-catenin-1* animals (**Figure 5**, 68/68). These results suggest that we successfully inhibited *DjAtg7* expression and that our RNAi method was valid. After 10 days of regeneration, RNAi-animals were fed, and the newly regenerated intestinal branches were filled with food (**Supplementary Figure S3**). Our results indicate that RNAi-*DjAtg7* does not only not affect planarian intestine regeneration, but that is also does not affect the physiological function of the intestinal system.

### Detection of Autophagosome Formation in RNAi-*DjAtg7* Animals

**FIGURE 7:**
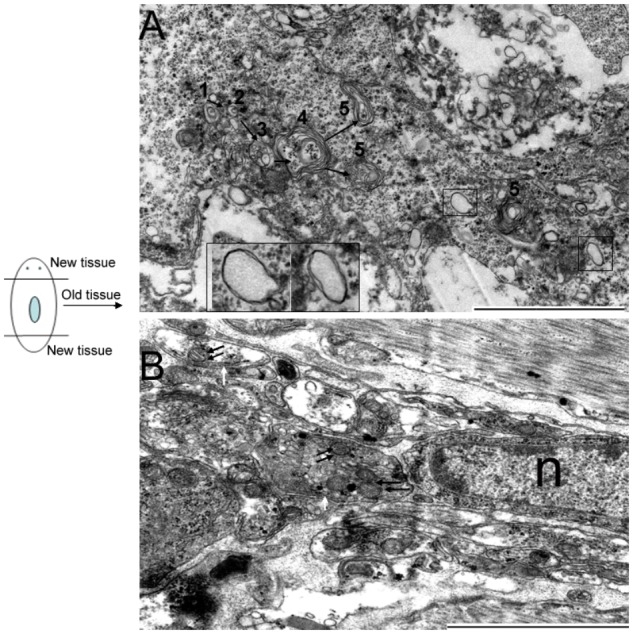
Autophagosome formation in RNAi-*DjAtg7* animals. Six samples were collected at 7 and 10 days of regeneration for TEM. **(A)** The process of autophagosome formation: (1) single-vesicle structure → (2) double-vesicle structure → (3) multiple-vesicle structure → (4) early autophagosomes with multiple membranes → (5) late autophagosome with multiple membranes. The black box indicates the double-membrane vesicle. **(B)** Mitochondria sequestered into double-membrane vesicles. The double black arrow indicates mitochondria. The white arrow indicates the double-membrane. n: nucleus; scale bar: 2 μm.

*DjAtg7* expression knockdown did not obviously affect planarian regeneration; therefore, we were interested in determining whether its knockdown blocked autophagosome formation in RNAi-animals. We observed that many cells in the old tissues exhibited autophagic features following amputation. **Figure 7A** shows the process of autophagosome formation (single-vesicle structure → double-vesicle structure → multiple-vesicle structure → early autophagosomes with multiple membranes → late autophagosome with multiple membranes), and **Figure 7B** shows that mitochondria are sequestered into double-membrane vesicles. It should be noted that the phenomenon illustrated by **Figure 4** and **Figure 7** occurs in both RNAi-animals and non-RNAi animals (**Supplementary Figure S4** provides more autophagic-like vesicles). Therefore, our results indicate that *DjAtg7* expression knockdown did not influence autophagosome formation.

### Detecting Atg Expression Levels in RNAi-*DjAtg7* Animals

**FIGURE 8:**
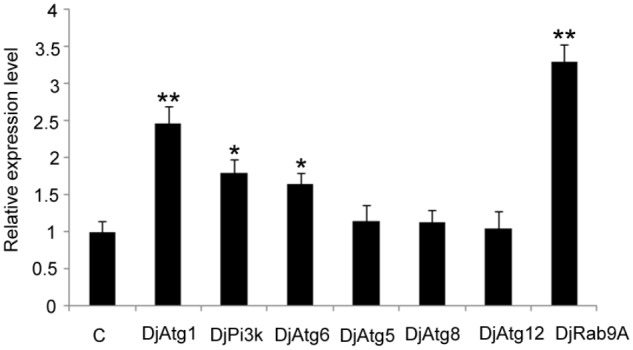
qPCR showing the relative expression level of Atgs in RNAi-*DjAtg7* animals. Shown are averages of three independent experiments; error bars = SEM, ^∗^*P* < 0.05, ^∗∗^*P* < 0.01 (Student’s *t*-test). Asterisks indicate significant differences compared to the control. Samples were collected at day 10 of regeneration.

Considering that Atg7-independent autophagy occurs in many organisms (Komatsu et al., 2005; Nishida et al., 2009), we suspected that this alternative pathway might also be present in planarians. Thus, we checked the expression levels of several molecules involved in conventional autophagy and alternative autophagy. The results showed that the expression levels of *DjAtg5*, *DjAtg8*, and *DjAtg12* did not change significantly in the RNAi-*DjAtg7* animals when compared to the control animals (**Figure 8**). However, *DjAtg1*, *DjPi3k*, and *DjAtg6* were upregulated in the RNAi-*DjAtg7* animals (**Figure 8**), and, in particular, the expression of *DjRab9A* increased by 3.3-fold in RNAi-*DjAtg7* animals (**Figure 8**). These results suggest that RNAi-*DjAtg7* activated the alternative autophagy pathway.

## Discussion

Planarians undergo continuous body size remodeling under prolonged starvation or following amputation, and this process may involve autophagy. Since González-Estévez proposed the use of planarians as ideal model organisms to study autophagy *in vivo* 10 years ago, limited studies on autophagy have been performed at a molecular level. Autophagy is currently known to be driven by >35 Atgs conserved from yeasts to mammals (He and Klionsky, 2009; Mizushima et al., 2011; Lee et al., 2015). Among these genes, *Atg7* is essential for autophagosome formation (Komatsu et al., 2005; Mortensen et al., 2011). No *Atg7* homolog has been characterized in planarians, and the function of *Atg7* in planarian regeneration has remained unknown. In this study, we identified the complete cDNA of *Atg7* from *D. japonica* for the first time. The full-length cDNA of *DjAtg7* measures 2272 bp and encodes a polypeptide of 693 amino acids. The deduced amino acid sequence of *DjAtg7* shares the conserved domains of the ATG7 superfamily: the putative ATP-binding domain (GxGxxG motif) and the catalytic domain with a conserved active center containing a cysteine residue (Nolting et al., 2009). The characteristics of the deduced amino acid sequences indicate that DjATG7 belongs to the family of protein-activating E1 enzymes (Nolting et al., 2009).

Atg7 is involved in energy metabolism (Karvela et al., 2016), cytoprotection (Xie et al., 2015; Zhuang et al., 2016), and stem cell maintenance (Gomez-Puerto et al., 2016). We aimed to determine whether the expression of *DjAtg7* is involved in planarian regeneration and body remodeling. In our study, the expression of *DjAtg7* gradually increased with regeneration, and its transcripts were mainly expressed in intestinal tissues of the intact animals at a basic level. Interestingly, *DjAtg7* was highly expressed at the newly regenerated intestinal branch during days 3–7 of regeneration (**Figure 3B**). This finding is consistent with the observation that planarians regenerate intestinal polarity at 3–7 days after amputation (Forsthoefel et al., 2011). Forsthoefel et al. (2011) also proposed that planarian intestine regeneration is involved in the remodeling of newly differentiated intestinal tissues and pre-existing tissues, which is consistent with the function of autophagy (Varga et al., 2014; Saera-Vila et al., 2016). *DjAtg7* transcripts were highly expressed in the newly regenerated intestinal branch and decreased to normal levels following the completion of regeneration, suggesting that the upregulation of *DjAtg7* in the blastema is related to reestablishing the function and morphology of the newly regenerated intestinal branches. We also found that *DjAtg7* was highly expressed in the distal branch of the old intestine system following the completion of regeneration (**Figure 3A**). This expression pattern suggests that *DjAtg7* is involved in body remodeling. For trunk fragment regeneration, when a new head and new tail are formed, a new planarian reshapes its body size; that is, the old tissue becomes narrow and lengthens. For planarian body remodeling, intestinal tissue atrophy should first occur at the distal branch. This speculation is consistent with the expression pattern of *DjAtg7* in the distal branch of the old tissue. Considering that planarian regeneration occurs under starvation conditions and that autodigestion of the intestinal tissues is a survival strategy for most animals exposed to starvation conditions (Sakiyama et al., 2009; Zeng et al., 2012; Funes et al., 2014; do Nascimento et al., 2016; Shan et al., 2016), we infer that autophagy that occurs in the distal branch of the old intestinal tissues can provide energy for planarian regeneration. This inference is supported by the recent findings that specific inhibition of autophagy genes in the intestine is sufficient to shorten the lifespan in dietary-restricted *Caenorhabditis elegans* (Gelino et al., 2016).

We used RNAi to inhibit *DjAtg7* expression and to further assess the biological function of this gene in planarian regeneration. Contrary to our expectations, no significant phenotype alteration was observed in the RNAi-animals (**Figure 5**), although endogenous *DjAtg7* mRNA was diminished to low levels during regeneration (**Figure 6**). We further verified that knocking down *DjAtg7* did not block autophagosome formation (**Figure 7**). In *D. melanogaster*, *Atg7* mutants are fully viable and fertile, and no major morphological defects are observed (Juhász et al., 2007). In filamentous ascomycetes, heterokaryotic Δ*Smatg7*/*Smatg7* mutants also display no alterations in their vegetative phenotype compared with that of the wild-type under non-starvation conditions, although the transcript levels of *SmAtg7* are drastically reduced (Nolting et al., 2009). Deletion of Atg7 in the intestinal epithelium is also dispensable for gut homeostasis (Wittkopf et al., 2012). Many researchers proposed the existence of Atg5/Atg7-independent autophagy in many organisms (Shimizu et al., 2010; Arakawa et al., 2017; Shimizu, 2018). Therefore, we suspected that Atg7-independent autophagy might occur in planarians. To test this hypothesis, we checked the molecules related to conventional and alternative autophagy. *Atg5*, *Atg8*, and *Atg12* are essential for conventional autophagy (Nishida et al., 2009; Arakawa et al., 2017), and their homologs are unaffected by RNAi-*DjAtg7*. *Atg1*, *Pi3k*, and *Atg6* are crucial to both types of autophagy (Nishida et al., 2009; Arakawa et al., 2017), and the expression levels of their homologs are apparently increased in RNAi-*DjAtg7* animals. RAB9 is required for the alternative autophagy pathway (Nishida et al., 2009; Arakawa et al., 2017). As expected, the expression level of *DjRab9A* increased significantly in the RNAi-*DjAtg7* animals, which suggested that an Atg7-independent autophagy pathway may exist in planarians. Considering that multiple *Atg* genes participate in conventional as well as other types of planarian autophagy (chaperone-mediated autophagy and Atg7-independent autophagy), we can conclude that specific inhibition of *DjAtg7* expression is not sufficient to affect planarian regeneration.

Though many reviews have addressed the importance of autophagy in planarian body remodeling (González-Estévez et al., 2007; González-Estévez, 2008, 2009; Felix et al., 2018), little morphological evidences exist to support this hypothesis. In this study, we not only observed autophagy occurring in the old tissue during planarian regeneration, we also observed a large number of cells exhibiting the features of autophagic cell death. Autophagic cell death is morphologically defined (especially by TEM) as a type of cell death that occurs in the absence of chromatin condensation but is accompanied by large-scale autophagic vacuolization of the cytoplasm (Levine and Yuan, 2005; Kroemer and Levine, 2008). Our observation is consistent with this definition. More interestingly, we observed a large number of cells in which the cytoplasm had been emptied, and we also observed vesicles being released through the plasma membrane (**Figure 4H**). Therefore, we infer that dying cells can provide nutrient materials for other cells (such as the neoblasts) through the release of vesicles containing cellular cytoplasmic contents into the extracellular space. This interpretation adequately explains our observation of many cells in which the cytoplasm had been emptied. We also observed that even the debris of the dead cells was sequestered in multilayered-membrane vesicles (**Figure 4G**), which may prevent inflammation and enable easy recycling of cellular components. Therefore, autophagy and autophagic cell death that occurred in the old tissue not only meet the demand for body remodeling but also meet the demand for energy supply during planarian regeneration.

In this work and for the first time, we successfully cloned the full-length cDNA of *Atg7* in *D. japonica* and analyzed its expression pattern during planarian regeneration. Our results reveal that *DjAtg7* is highly expressed at the newly regenerated intestinal branch at the early stage of planarian regeneration and at the distal branch of the intestine system at the late stage of planarian regeneration. Our results also show that *DjAtg7* knockdown by RNAi does not affect planarian regeneration and autophagosome formation. These findings indicate that planarian autophagy is more complex than previously realized. Our TEM observation clearly confirms that autophagy and autophagic cell death occur in the old tissues of the newly regenerated planarians. Collectively, our work provides important data for the understanding of autophagy and autophagic cell death associated with planarian regeneration and body remodeling.

## Ethics Statement

This study was carried out in accordance with ethical standards.

## Author Contributions

KM acquired the data, analyzed, and prepared the manuscript. YZ and GS acquired the data. MW prepared the materials. KM and GC conceived and designed the study. GC approved the final manuscript.

## Conflict of Interest Statement

The authors declare that the research was conducted in the absence of any commercial or financial relationships that could be construed as a potential conflict of interest.
