# Supplementary material for: Identification of Autophagy-Related Gene 7 and Autophagic Cell Death in the Planarian Dugesia japonica
Source: Front Physiol. 2018 Sep 4;9:1223. doi: 10.3389/fphys.2018.01223 (PMC6131670; doi:10.3389/fphys.2018.01223)

**Supplementary figure legends**

S1 qPCR analysis of *DjAtg7* expression levels on different days following the amputation. Shown are averages of three independent experiments; error bars = SEM, *P < 0.05 (Student’s *t*-test). Asterisks indicate significant differences compared to the control. The planarians collected at time zero are cut and used as the control.

S2 Double FISH of *DjAtg7* (red) and *DjPk-1/DjWi-1* (green) with DAPI (blue). Intense signal of *DjAtg7* (red) shows the newly regenerated intestinal branch in the tail, and which is not merged with the *DjWi-1* (green) signal. n﹦6 animals. Scale bars: 200 μm.

S3 Animals were fed after 10 days of regeneration. There is no obvious difference between the RNAi- and the non-RNAi-animals in the newly regenerated intestinal branches. The broken brown line represents the border between blastema and old tissue. n﹦20 animals. Scale bar 1 mm.

S4 Different stage of autophagosomes. A: showing the autophagosome containing the cytoplasmic content. B: showing a autolysosome with partially digested cytoplasmic content. C-D: showing the residual bodies. Scale bar 2 μm.

Supplementary figures

**S1**


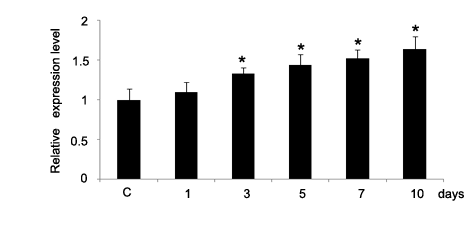


**S2**


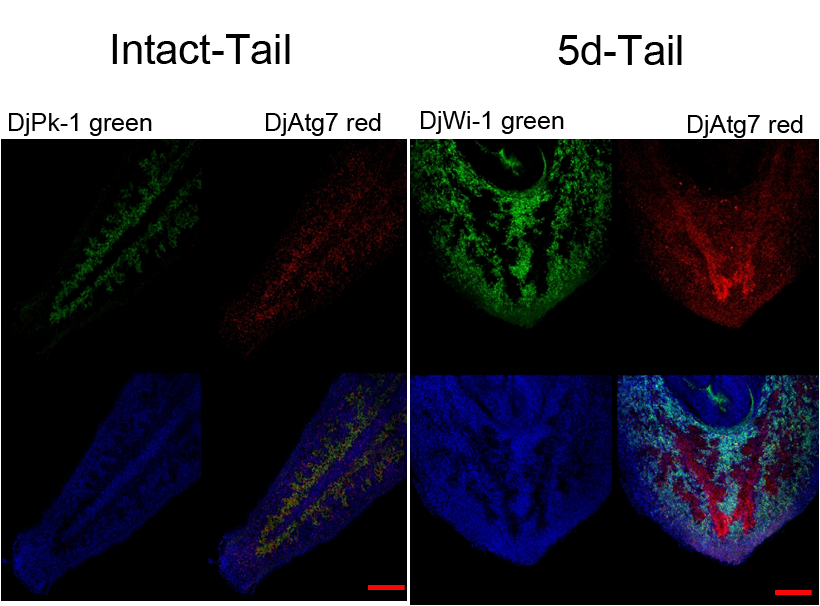


**S3**


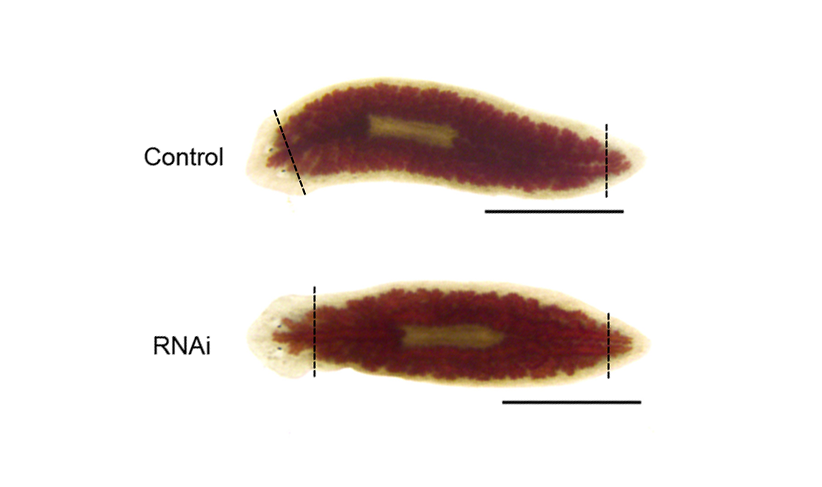


**S4**


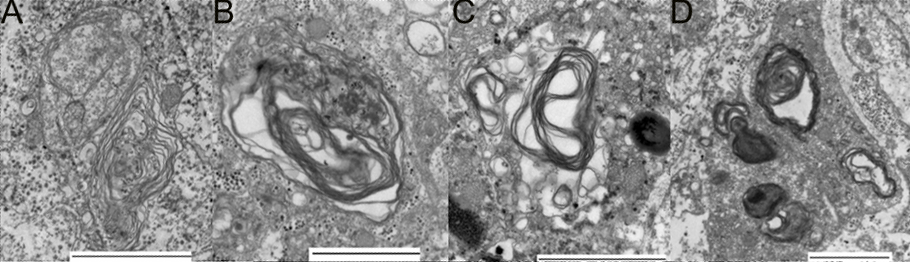

Supplement: Supplementary file 1 [file Data_Sheet_1.doc]
